# Characteristics and dynamics of malaria vectors around the Soum dam in Nanoro Health District, Burkina Faso

**DOI:** 10.1371/journal.pone.0348192

**Published:** 2026-04-30

**Authors:** Hamidou Ilboudo, Elodie Doda Gricela Sanon, Domonbabele François de Sales Hien, Fadilah Traoré, Helle Hansson, Bérenger Kaboré, Marc Christian Tahita, Karim Derra, Eli Rouamba, Ismaïla Bouda, Toussaint Rouamba, Michael Alifrangis, Hermann Sorgho, Pascal Magnussen, Halidou Tinto

**Affiliations:** 1 Institut de Recherche en Sciences de la Santé/Unité de Recherche Clinique de Nanoro, Nanoro, Burkina Faso; 2 Institut de Recherche en Sciences de la Santé/Direction Régionale de l’Ouest, Burkina Faso; 3 Centre for translational Medicine & Parasitology, Department of Immunology and Microbiology, Copenhagen, Denmark; 4 Department of Infectious Diseases, Copenhagen University Hospital, Copenhagen, Denmark; National Institute of Malaria Research, INDIA

## Abstract

Malaria is endemic in Burkina Faso, with seasonal transmission during the rainy season. Environmental changes such as dam construction may influence mosquito ecology and malaria transmission; however, entomological data from this area remain limited. This study aimed to characterize malaria vector dynamics, including species composition, blood meal sources, and sporozoite infection rates, in five villages at varying distances from the Soum dam located in the Nanoro Health District catchment area in Burkina Faso. From March 2022 to February 2023, mosquitoes were collected monthly via pyrethrum spray catches (PSCs) targeting indoor resting vectors. Mosquitoes were identified morphologically via taxonomic keys. PCR analyses were performed to identify species within the *Anopheles gambiae* complex, determine blood meal sources, and assess *Plasmodium falciparum* infection. A total of 11,378 *Anopheles* mosquitoes were collected, including 3,432 males (30.1%) and 7,948 females (69.9%). *An. gambiae s.l*. was the most abundant species (86.5%), followed by *An. funestus* (10.6%). Within the *An. gambiae* complex, 91.5% were *An. coluzzii*, 8.3% *An. arabiensis*, and 0.2% *An. gambiae sensu stricto*. The vector density was highest in Soum (49.7%) and decreased with distance from the dam. The overall sporozoite rate was 6.2%, with higher rates in Seguedin (9.5%) and Soala (8.7%). Among the tested mosquitoes, 34.7% fed on humans, 14.2% on animals, and 23.6% on both. *Anopheles coluzzii* was the predominant vector and showed moderate anthropophilic behavior. Despite higher vector density near the dam, infection rates were greater in distant villages, highlighting the complexity of vector dynamics in dam-associated areas and the need for localized control strategies.

## Background

Malaria is an infectious disease caused by parasites of the genus *Plasmodium* and is transmitted to humans through the bites of infected female mosquitoes of the genus *Anopheles* [1]. It remains a major public health problem, with 263 million cases and 597,000 deaths reported worldwide in 2023, of which approximately 95% of cases and 96% of deaths occurred in sub-Saharan Africa [2].

In Burkina Faso, malaria remains the leading cause of outpatient consultations, hospitalizations, and deaths in health facilities, with more than 12 million cases recorded in 2021, including 4,355 deaths [3]. Pregnant women and children under five years of age are the most vulnerable groups [4]. Although malaria is endemic throughout the country, transmission peaks occur during the rainy season (June to October), placing the entire population at risk.

Several *Anopheles* species are involved in malaria transmission, with *Anopheles gambiae* (*An. gambiae*) complex being the most widespread and epidemiologically significant across sub-Saharan Africa [5,6]. This complex comprises eight morphologically similar species [7], among which *An. gambiae sensu stricto* (*An. gambiae s.s*.), *An. coluzzii*, and *An. arabiensis* are highly anthropophilic and serve as the primary vectors of *Plasmodium falciparum* (*P. falciparum*), the parasite responsible for most malaria-related deaths in the region [2,8].

In the context of climate change, Burkina Faso has experienced reduced rainfall and rising average temperatures over recent decades [9]. To address food insecurity and promote agricultural development, several agropoles have been created around artificial dams [10,11]; one of them being the Soum agropole, which was established between 2003 and 2013 around the Soum dam in the Nanoro Health District (NHD) and is intended to increase local irrigation and food production.

However, the presence of permanent and semi-permanent water bodies linked to dam infrastructure can promote mosquito breeding and alter vector population dynamics. This may, in turn, increase malaria transmission risk by sustaining vector presence beyond the typical transmission season. However, little is known about how proximity to the Soum dam could affect vector ecology and transmission parameters.

This study aimed to assess the characteristics of malaria vectors, including their species composition, abundance, blood meal sources, and infection rates with *P. falciparum*, in five villages situated at varying distances from the Soum dam.

## Materials and methods

### Study area

This study was conducted in the NHD catchment area, which is located in the Centre-West region of Burkina Faso at about 87 km from Ouagadougou, the capital city. The NHD has an estimated population of 166,683 and includes 26 peripheral health centres (PHCs), 4 medical centres (MCs) and one referral district hospital (CMA, Saint Camille de Nanoro). The NHD is located in the Sudano-Sahelian climate zone, which is characterized by two main seasons: a rainy season from June to October (average rainfall of 450–700 mm/year, average temperature >30°C) and a dry season from November to May (temperature between 17–43°C). Mosquito collections were carried out in five villages located at varying distances from the Soum dam: Soum (0 km), where the dam is situated; Goulouré (8 km); Nazoanga (14 km); Seguedin (33 km); and Soala (40 km).

### Study design and mosquito collection

In each village, five households were randomly selected every month for mosquito collection, with different houses chosen on each occasion. Mosquito sampling was conducted once per month in each village. Collections were carried out on a single day per village, typically during the same week at each month, although not always on the same calendar date. All five villages were sampled within the same week to ensure comparability across sites. Mosquito sampling was conducted from March 2022 to February 2023 via pyrethrum spray catches (PSCs), which target indoor resting mosquitoes. Collections were carried out early in the morning between 06:00 and 08:00. The collected mosquitoes were identified using morphological criteria under a binocular loupe, following the identification key of Gillies and Coetzee (1987) [12]. The samples were then sorted by sex. Female *Anopheles* mosquitoes were individually stored in 1.5 mL Eppendorf tubes containing silica gel and preserved at −20 °C for subsequent molecular analysis. Male *Anopheles* were counted, identified, and discarded. In addition to *Anopheles*, culicine mosquitoes (mainly *Culex* spp. and *Aedes* spp.) were also collected. *Culex* were recorded and discarded, while *Aedes* were preserved separately for potential future analyses. However, only *Anopheles* mosquitoes were included in the present study.

### Sampling for molecular analysis

For molecular analyses, up to 20 female *Anopheles* mosquitoes were randomly selected per village per month. The specimens were preferentially selected among blood-fed and semi-gravid females, followed by unfed individuals when necessary. Selection was based on the availability of morphologically identified female specimens from each monthly sample collection. In several instances, particularly during the dry season, the number of available females was insufficient to reach this threshold because of low mosquito densities (Table 1). As a result, a total of 995 samples were analysed across all villages and time points.

**Table 1:** Numbers of female *Anopheles gambiae s.l*. mosquitoes collected per month.

	Soum	Goulouré	Nazoanga	Seguendin	Soala	Total N (%)
March 2022	39	24	16	12	13	**104** (1.5)
April 2022	32	5	0	1	1	**39** (0.6)
May 2022	142	21	12	3	6	**184** (2.7)
June 2022	267	22	4	2	28	**323** (4.7)
July 2022	407	84	180	66	135	**872** (12.7)
August 2022	142	151	323	64	169	**849** (12.3)
September 2022	1234	386	308	98	170	**2196** (31.9)
October 2022	845	573	110	174	34	**1736** (25.3)
November 2022	53	39	56	36	11	**195** (2.8)
December 2022	61	59	58	12	29	**219** (3.2)
January 2023	20	3	5	8	0	**36** (0.5)
February 2023	33	1	79	0	9	**122** (1.8)
**Total**	**3275**	**1368**	**1151**	**476**	**605**	**6875 (100)**

### DNA extraction

DNA was extracted from the abdomens of blood-fed and semi-gravid females for the identification of blood meal sources and from the head-thorax of female mosquitoes for the detection of *P. falciparum* sporozoites. The extraction was performed using the 2% cetyltrimethyl ammonium bromide (CTAB) extraction protocol described by Morlais et al. (2004) [13]. The purified DNA was diluted in 20 µl of molecular biology-grade water and stored at −20°C for subsequent analysis.

### Molecular analysis

Molecular analyses were performed to identify sibling species within the *Anopheles gambiae* complex, determine blood meal sources, and detect *P. falciparum* sporozoite infections. Species identification used the SINE200 (Short Interspersed Nuclear Element 200) PCR assay, a molecular marker consisting of a 200 bp repetitive DNA sequence on the *An. gambiae* X chromosome that differentiates sibling species [14]. Blood meal sources were determined using two multiplex PCR assays targeting the mitochondrial cytochrome b (cyt b) gene as described in [15], targeting human, pig, cow, dog, chicken, goat, sheep, and donkey hosts. Detection of *P. falciparum* sporozoites was performed by PCR following the protocol in [16].

All primer sequences, expected amplicon sizes, representative gel images, and datasets supporting these analyses are provided in the Supporting Information files.

### Statistical analysis

Descriptive statistics were used to summarize the mosquito species composition and sex ratios across villages and collection months. The human blood index (HBI) was calculated as the proportion of blood-fed mosquitoes containing human blood, with 95% confidence intervals (CIs), using exact binomial statistics.

To assess significant differences in mosquito abundance and infection rates across sites and time periods, chi-square tests were used to compare categorical variables. When expected cell counts were less than five, Fisher’s exact tests were applied. All the statistical analyses were performed in STATA version 16.0, with significance defined as p < 0.05.

### Ethics statement

This study is part of the CLIMSA project (Mitigating Climate Change on Health in Burkina Faso), for which the study protocol was approved by the “Comité d’Éthique pour la Recherche en Santé” (CERS: N°2021/12/273). No human or animal volunteers were involved in the entomological surveys. Community members were informed about the study objectives, and oral informed consent to use their homes for mosquito collection was obtained from household heads in the presence of a community guide who served as a witness. Field activities were conducted with authorization from the Nanoro Health District authorities and local village leaders; no additional permits were required.

## Results

### *Anopheles* mosquito species composition

A total of 11,378 *Anopheles* mosquitoes were collected (3,432 (30.1%) males and 7,948 females (69.9%), resulting in a female-to-male sex ratio of 2.3:1. Since only female mosquitoes transmit malaria, the species composition analysis focused on them. *Anopheles gambiae s.l*. was the most abundant species collected in all villages (n = 6,875; 86.5%), followed by *An. funestus* group (n = 839; 10.6%) and *An. rufipes* (n = 218; 2.7%). A few *An. flavicosta* (n = 14; 0.2%) and *An. pharoensis* (n = 2; 0.02%) were also recorded.

**Fig 1:**
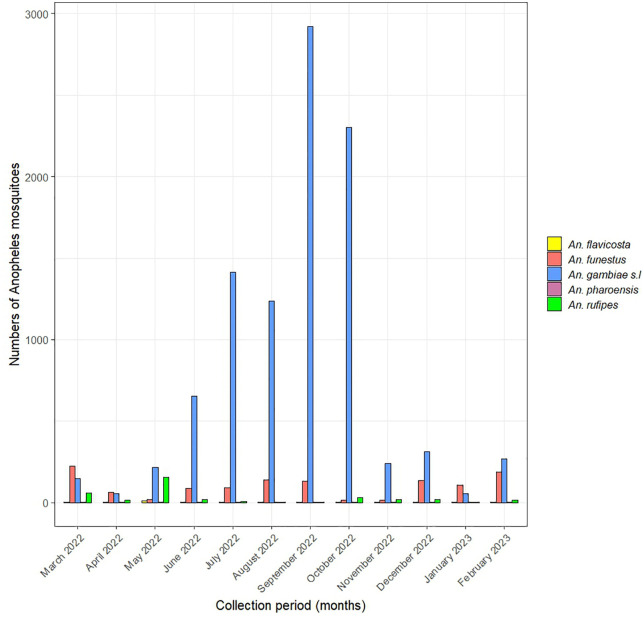
Temporal dynamics of *Anopheles* mosquito species collected over one year.

The abundance of female *Anopheles* differed significantly across villages (χ² = 6117.5, p < 0.001), with the highest density being recorded in Soum (n = 3,951; 49.7%), followed by Goulouré (n = 1,566; 19.7%), Nazoanga (n = 1,309; 16.5%), Soala (n = 624; 7.9%), and Seguedin (n = 498; 6.2%) (Table 2). *Anopheles gambiae s.l*. was collected throughout the year, with markedly higher abundances during the rainy season (June to October). The peak abundance was observed in September (Fig 1).

**Table 2:** Species composition of *Anopheles* mosquitoes collected from 5 villages over the 12 months period.

Species	Soum	Goulouré	Nazoanga	Seguendin	Soala	Total n (%)
*An. gambiae s.l*.	3 275	1 368	1 151	476	605	6 875 (86.5)
*An. funestus*	496	179	138	18	8	839 (10.6)
*An. rufipes*	167	19	17	4	11	218 (2.7)
*An. flavicosta*	12	0	2	0	0	14 (0.2)
*An. pharoensis*	1	0	1	0	0	2 (0.0)
Total	**3 951**	**1 566**	**1 309**	**498**	**624**	**7 948 (100)**

*Anopheles funestus* abundance also differed significantly between villages (χ² = 1167.3, p < 0.001), with the highest density being recorded in Soum (n = 496; 59.1%), followed by Goulouré (n = 179; 21.3%), Nazoanga (n = 138; 16.4%), Seguedin (n = 18; 2.1%), and Soala (n = 8; 1.0%).

### Molecular characterization of *An. gambiae* complex

Using the SINE200 PCR assay, 848 (85.2%) of the 995 *An. gambiae s.l*. mosquitoes analysed were successfully identified to the species level. *Anopheles coluzzii* was the most predominant species (91.5%), followed by *An. arabiensis* (8.3%) and *An. gambiae* s.s. (0.2%).

**Fig 2:**
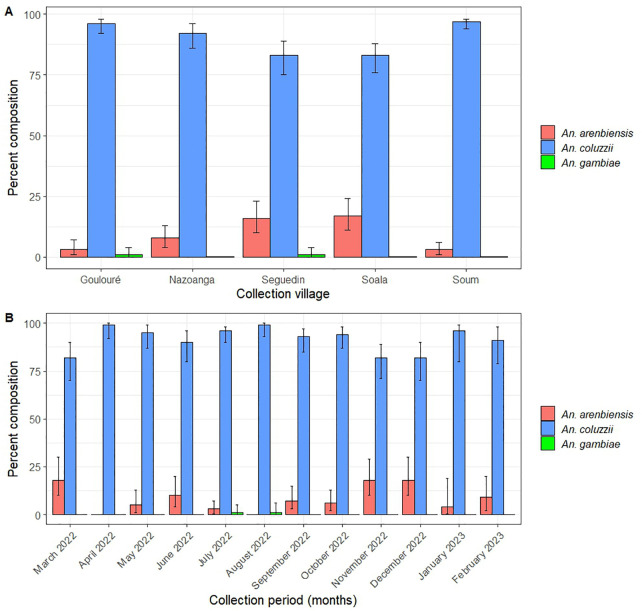
Spatial and temporal variations in *An. gambiae s.l.* composition. A: Species distribution of *An. gambiae* complex by village. B: Monthly variation in *An. gambiae* species complex composition.

*Anopheles coluzzii* represented more than 80% of the identified *An. gambiae* complex mosquitoes in all five villages (Fig 2A). *Anopheles arabiensis* was also present at varying proportions: 16.6% in Soala, 16.5% in Seguedin, 8.0% in Nazoanga, 3.3% in Goulouré, and 2.6% in Soum (Fig 2A). *Anopheles gambiae* s.s. was detected at a very low proportion of 0.7% and 0.8% in only Goulouré and Seguedin respectively.

*Anopheles coluzzii* was consistently present throughout the year and represented more than 80% of the species. *Anopheles arabiensis* was significantly more prevalent during the dry season than in the rainy season (χ² = 8.60, p = 0.003) (Fig 2B).

**Blood meal sources of mosquitoes.**

**Fig 3:**
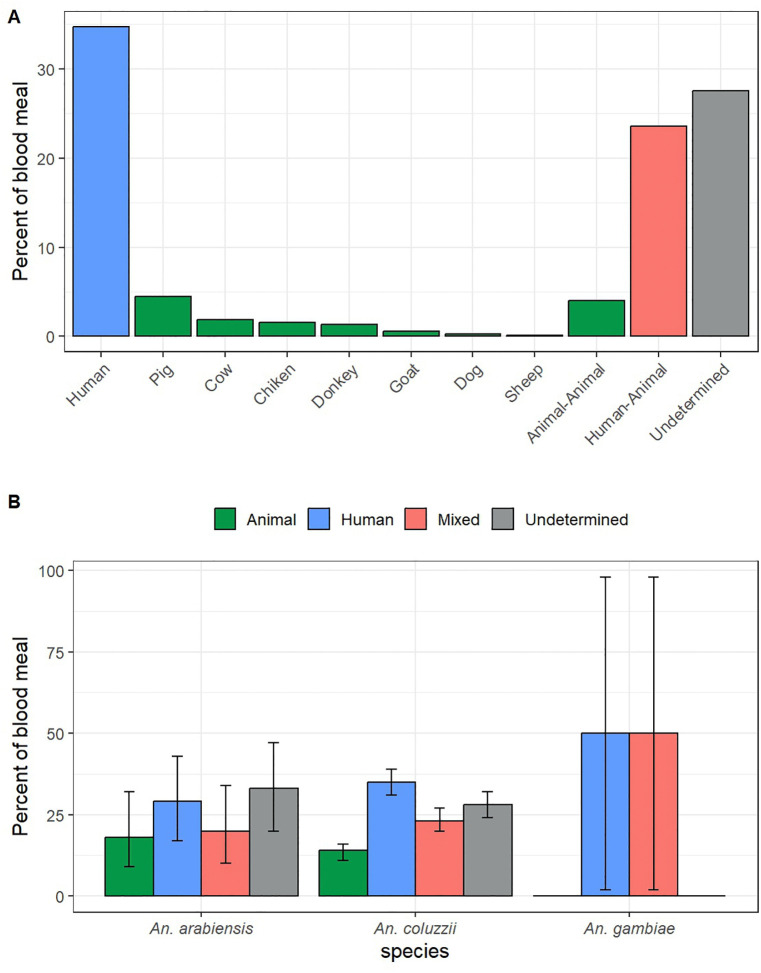
Blood meal sources of *An. gambiae s.l.* (A) Proportions of human, animal, mixed, and unidentified blood meals among all tested *Anopheles* mosquitoes. (B) Blood meal origin by species within the *An. gambiae s.l*.

A total of 755 blood-fed and semi-gravid *Anopheles* mosquitoes were tested for blood meal origin by cytochrome (cyt) b multiplex PCR. Among these, 262 (34.7%) fed human blood, 107 (14.2%) fed animal blood, 178 (23.6%) fed both human blood and animal blood, and 208 (27.5%) were tested negative for all targeted hosts (Fig 3A). Among the animal-derived blood meals, pigs were the most common host (4.5%), followed by cows (1.9%), chickens (1.6%), goats (0.5%), dogs (0.3%), and sheep (0.1%).

Within the *An. gambiae* complex, 35.2% of *An. coluzzii* fed on human blood, 13.6% on animal blood, and 23.3% on both. For *An. arabiensis*, 28.6% fed on human blood, 18.4% on animal blood and 20.4% on both. (Fig 3B). Out of the two *An. gambiae* s.s. specimens tested, one fed on human blood and the other on a mixed blood meal (Fig 3B).

### *Plasmodium falciparum* sporozoite infection

**Fig 4:**
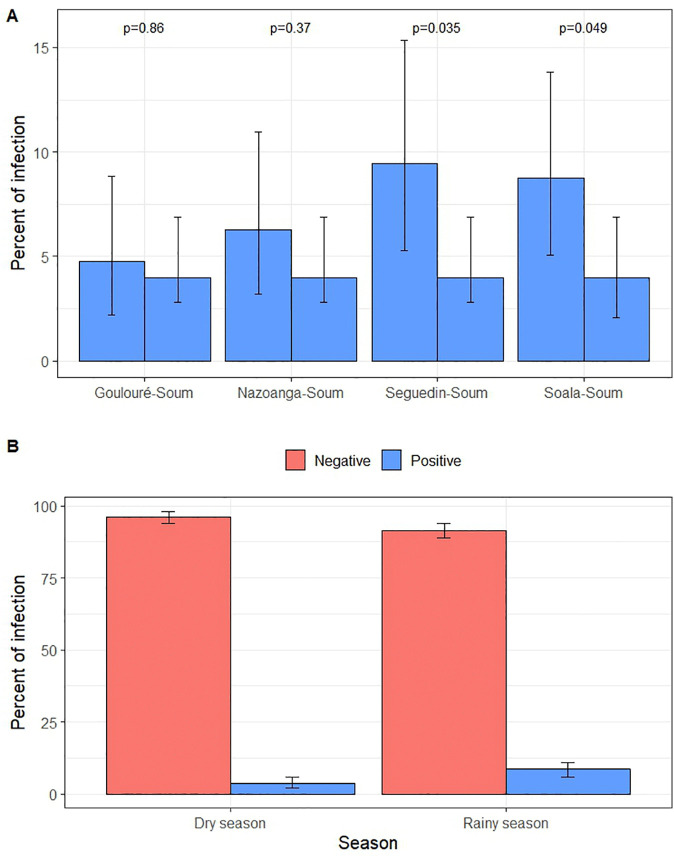
*P. falciparum* sporozoite infection in *An. gambiae s.l.* mosquitoes. A: Infection rates by village. B: Infection rates by season (dry and rainy).

A total of 995 *An. gambiae* complex mosquitoes were tested for *P. falciparum* infection and were distributed as follows: Soum (n = 300), Goulouré (n = 189), Nazoanga (n = 175), Seguedin (n = 148), and Soala (n = 183). The overall sporozoite infection rate was 6.2% (95% CI: 4.8–7.9). The latter varied across the five villages: 9.5% in Seguedin (95% CI: 5.3–15.4), 8.7% in Soala (95% CI: 5.1–13.8), 6.3% in Nazoanga (95% CI: 3.2–11.0), 4.8% in Goulouré (95% CI: 2.2–8.9), and 4.0% in Soum (95% CI: 2.2–7.2) (Fig 4A). Pairwise comparisons revealed significantly higher infection rates in Seguedin than in Soum (χ² = 4.45, p = 0.035) and Soala (χ² = 3.85, p = 0.049). A seasonal variation was also observed: the infection rate during the rainy season was 8.5% (95% CI: 6.3–11.3), whereas it was 3.8% (95% CI: 2.2–5.9) in the dry season (χ² = 8.87, p = 0.003) (Fig 4B).

## Discussion

This study showed that the malaria vectors in the study area were predominantly *An. gambiae s.l*., particularly *An. coluzzii*, whereas *An. funestus* was present at a very low rate. The ecological characteristics of the area, especially the absence of permanent water bodies with vegetation, may explain the low abundance of *An. funestus* [17], which prefers such habitats. However, the spatial distribution of *An. funestus* is more common in villages closer to the Soum dam (Soum, Goulouré, Nazoanga). This may reflect the role of the dam in creating localized habitats favourable to this species, such as slow-moving irrigation channels or permanent water bodies with emergent vegetation. These observations suggest that, despite broader ecological shifts, localized microhabitats can still support *An. funestus* populations within irrigated landscapes. Conversely, the irrigated landscape around the Soum dam, marked by rice paddies and market gardens, offers abundant sunlit, shallow, and semi-permanent water bodies that are highly favourable for *An. coluzzii.* This likely explains its predominance in villages near the dam. These findings are consistent with observations from other irrigated zones in East and West Africa, where the construction of large dams and the development of cultivated land have been associated with increased malaria vector proliferation [18–20].

However, the study also revealed an intriguing paradox: villages farther from the dam, such as Seguedin and Soala, presented lower mosquito numbers but higher sporozoite infection rates. A first plausible explanation is that mosquito longevity may be greater in these remote villages, where ecological conditions, such as lower human activity, may favour mosquito survival. Longer-lived mosquitoes are more likely to become infectious, which could explain the higher infection rates despite the lower overall abundance of mosquitoes. In contrast, proximity to dams in villages such as Soum may result in the rapid and continuous emergence of mosquitoes due to an abundance of breeding sites, but the average mosquito population is relatively young. This would result in higher densities but a lower infection rate. Previous studies have also shown that despite high mosquito densities in Vallée du Kou, in Burkina Faso, the sporozoite rate was low [21]. In addition, the infection rate of mosquitoes infected with *P. falciparum* was greater during the rainy season than in the dry season. This may be explained by the higher prevalence of malaria during the rainy season, which increases the number of parasites infecting mosquitoes.

The analysis of mosquito feeding behaviour revealed a strong anthropophilic tendency among *An. gambiae* complex species. This aligns with the known behavioural profile of *An. coluzzii*, which tends to rest indoors and preferentially feeds on humans [22,23]. Overall, the HBI observed in this study supports the occurrence of frequent human‒vector contact, highlighting the importance of effective personal protection measures, such as insecticide-treated nets (ITNs). The mixed blood meals and zoophilic profiles observed may indicate opportunistic feeding behaviour, particularly when humans are less accessible or when alternative hosts are abundant [24].

Another important observation concerns the 27.6% of the blood-fed mosquitoes reported for which no host DNA was detected. This could be attributed to low or degraded blood content in the samples or to feeding on untested animal species. The absence of DNA quantification and the limited host panel are acknowledged as limitations of blood meal analysis.

The study limitations should also be acknowledged. Molecular analyses were based on a maximum of 20 mosquitoes per village per month. While this standardized approach ensures balanced sampling across space and time, it may have limited the ability to detect low-frequency events such as sporozoite infections or blood meals from rare hosts. Increasing the sample size, especially in high-density periods or sites, would have contributed to improving the statistical power to detect differences in infection rates and host preference and then, enhance inference on spatial and temporal patterns.

## Conclusion

This study confirms the strong influence of dam infrastructure and associated agricultural practices on the ecology of malaria vectors in the Nanoro Health District. However, it also shows that increased vector abundance near the dam does not necessarily correspond to increased malaria transmission. Infection risk appears to be shaped by a combination of ecological factors, vector survival, and human-vector interactions. These findings highlight the importance of integrated vector management strategies that combine entomological surveillance, environmental planning, and targeted public health interventions in dam-affected regions.

## Supporting information

S1 Fig Agarose gel electrophoresis for species identification in *Anopheles gambiae s.l*. mosquitoes. Lane 1: molecular weight marker (100 bp ladder); Lane 2: *An. coluzzii* (479 bp); Lane 3: *An. gambiae* (249 bp); Lanes 4–5: *An. arabiensis* (223 bp).
(TIF)

S2 Fig Agarose gel electrophoresis for blood meal source identification in *Anopheles gambiae s.l*. mosquitoes. Lane 1: molecular weight marker (100 bp ladder); Lane 2: Pig (500 bp); Lane 3: Negative; Lane 4: Human (350 bp); Lane 5: Mixed Pig + Human (500 + 350 bp); Lane 6: Negative; Lane 7: Mixed Cow + Human (600 + 350 bp).
(TIF)

S3 Fig Agarose gel electrophoresis of PCR products for *Plasmodium falciparum* detection in *Anopheles gambiae s.l*. mosquitoes. Lanes 2, 3, 4, and 6: samples positive for *P. falciparum* (480 bp); Lane 1: negative sample; Lane 5: molecular weight marker (100 bp ladder).
(TIF)

S1 Table Primers used for PCR assays and expected amplicon sizes. (DOCX)

S1 Dataset Field mosquito collections around the Soum Dam. (XLSX)

S2 Dataset Molecular analysis results for Anopheles gambiae complex mosquitoes. (XLSX)
