# Supplementary material for: Characteristics and dynamics of malaria vectors around the Soum dam in Nanoro Health District, Burkina Faso
Source: PLoS One. 2026 Apr 30;21(4):e0348192. doi: 10.1371/journal.pone.0348192 (PMC13132233; doi:10.1371/journal.pone.0348192)
Supplement: S1 Table — (DOCX) [file pone.0348192.s004.docx]

**Supplementary table S1: Primers used for PCR assays and expected amplicon sizes**

| **PCR assay** | **Primer name(s)** | **Sequence (5’→3’)** | **Target species / host** | **Expected product size (bp)** | **Reference** |
| --- | --- | --- | --- | --- | --- |
| *An. gambiae* complex species ID | S200X6.1F / S200X6.1R | F: TCG-CCT-TAG-ACC-TTG-CGT-TA R: CGC-TTC-AAG-AAT-TCG-AGA-TAC | *An. coluzzii* / *An. gambiae s.s.* / *An. arabiensis* | 479 / 249 / 223 | Santolamazza et al., 2008 |
| Blood meal sources (Multiplex PCR 1) | Donkey 574F | CTG-GTA-ATC-GTC-CAT-CTA-C | Donkey | 460 | Vantaux et al., 2021 |
|  | Goat 894F | CCT-AAT-CTT-AGT-ACT-TGT-ACC-CTT-CCT-C | Goat | 150 | Vantaux et al., 2021 |
|  | Sheep 695F | CTA-TCC-TAC-TAA-TCC-TCA-TCC-TCA-TG | Sheep | 340 | Vantaux et al., 2021 |
|  | Chicken 470F | ACA-CAC-CCT-AGT-AGA-GTG-AGG | Chicken | 290 | Vantaux et al., 2021 |
|  | Rev1025 | GGT-TGT-CCT-CCA-ATT-CAT-GTT-A | Reverse primer (all above) |  | Vantaux et al., 2021 |
| Blood meal sources (Multiplex PCR 2) | Human 741F | GGC-TTA-CTT-CTC-TTC-ATT-CTC-TCC-T | Human | 350 | Vantaux et al., 2021 |
|  | Dog 365F | GGA-ATT-GTA-CTA-TTA-TTC-GCA-ACC-AT | Dog | 750 | Vantaux et al., 2021 |
|  | Cow 121F | CAT-CGG-CAC-AAA-TTT-AGT-CG | Cow | 600 | Vantaux et al., 2021 |
|  | Pig 375F | CCT-CGC-AGC-CGT-ACA-TCT-C | Pig | 500 | Vantaux et al., 2021 |
|  | Rev1025 | GGT-TGT-CCT-CCA-ATT-CAT-GTT-A | Reverse primer (all above) |  | Vantaux et al., 2021 |
| *P. falciparum* detection | Pf1 / Pf2 | F: GGA-ATG-TTA-TTG-CTA-ACA-C R: AAT-GAA-GAG-CTG-TGT-ATC | *P. falciparum* | 480 | Morassin et al., 2002 |
